# Authority and solidarity on the Estonian COVID-19 signs: In line with the government's guidelines, we ask you to wear a mask

**DOI:** 10.3389/frai.2022.1000188

**Published:** 2023-01-09

**Authors:** Ilona Tragel, Aimi Pikksaar

**Affiliations:** Department of General Linguistics, Institute of Estonian and General Linguistics, University of Tartu, Tartu, Estonia

**Keywords:** phatic communion, Estonian, grammatical person, imperative mood, authority, solidarity, COVID-19, public sign

## Abstract

This article presents the results of a quantitative analysis of 900 Estonian COVID-19 door signs, which were studied to investigate the linguistic means of establishing and maintaining contact between the sign's author (institution) and the addressee (client). Malinowski's notion of “phatic communion” and Laver's notions of “self-oriented” and “other-oriented” utterances as means for expressing status relations—authority and solidarity—between the participants of the communication act were used to establish four types of grammatical person usage on the COVID-19 signs: (1) “neither 1st nor 2nd person”; (2) “1st person only”; (3) “2nd person only”, and (4) “both 1st and 2nd person”. Grammatical person of personal pronouns and verb forms were included. The presence and absence of two other means for expressing authority—the imperative mood and lexical expressions of authority—were analyzed within these four types of grammatical person usage. The most important difference emerged between the signs belonging to the types “2nd person only” (i.e., signs with only other-oriented 2nd person, without 1st person) and “both 1st and 2nd person” (i.e., signs with both self-oriented 1st person and other-oriented 2nd person). On the signs belonging to the type “2nd person only” that, relying on Laver, express the higher status of the sender of the message in relation to the receiver of the message, the authors of the signs use significantly more imperative mood and less refer to an authority outside the communication act, thus putting themselves in the role of authority. However, on the signs belonging to the type “both 1st and 2nd person” that, relying on Laver, express the solidarity of the sender of the message with the addressee, the authors of the signs seem less inclined to assume the role of authority (using less imperative mood) and rather call the reader of the sign to submit to some higher authority (using lexical expressions of authority, e.g., *Vabariigi Valitsus* “Government of the Republic”, *Terviseamet* “Health Board”, etc.) to which the author of the sign and the addressee are both in a subordinate position and, therefore, of equal status.

## Introduction

We have recently lived in times of different kinds of social distancing—complete lockdown, keeping a 1.5–2 m distance, wearing a mask, etc. The government announced a state of emergency in Estonia on 12 March 2020, whereby various measures to combat the COVID-19 were implemented. These measures led to the temporary closure of many institutions. The situation and measures varied in different countries, but the main aim was to restrict face-to face communication between people to stop the spread of the virus. Public signs were one of the many means to deliver messages of closure or restrictions during the COVID-19 pandemic across the world.

The traditional framework for public signs' research has been Linguistic Landscape. This approach defines signs as “the linguistic items found in the public space” (Shohamy, 2006, p. 110), and as a form of asynchronous, one-way communication addressing unknown recipients (see, e.g., Shohamy, 2006; Barron, 2012; Blommaert, 2013). During the coronavirus pandemic, the global discourse emerged, which provided the unifying feature of the COVID-19 public signs: the setting, i.e., the situation where certain conditions are clearly established and even declared by the governments (e.g., the official declaration of the lockdown). Public messages of the pandemic could thus be studied as an example of crisis communication. In this field, there are a few studies of public signs of the crisis from a pre-COVID era (e.g., Tan and Said, 2015; Doroja-Cadiente and Valdez, 2019) and increasing amount of studies about COVID-19 signs (Kellaris et al., 2020; Li, 2020; Hua, 2021; Jing and Wang, 2021; Marshall, 2021; Ogiermann and Bella, 2021; Bella and Ogiermann, 2022; Dancygier et al., in press; Isosävi, in press). In addition to the different situations (crisis or non-crisis etc.) in which public signs are used, the perspective from which the analysis of the signs is conducted is also significant. Linguistic Landscape studies focus on multilingualism and/or interpret public signs as semiotic objects. There is significantly less research on the linguistic (lexical and/or grammatical) means used on public signs (from the pre-COVID era, e.g., Wierzbicka, 1998; Wetzel, 2010; Mautner, 2012; Wagner, 2015; Bonner, 2016; Ferenčík, 2018; Svennevig, 2021; and about the COVID-19 signs, e.g., Dancygier, 2021; Ogiermann and Bella, 2021; Bella and Ogiermann, 2022; Dancygier et al., in press). Our study contributes to the latter direction.

There are two important relations in the texts of the signs: interpersonal relations between the author and the addressee, and intertextual relations between the sign's text and other texts, e.g., regulations by the authority. What makes COVID-19 signs significant as a communication challenge is that the speech act performed by the sign implies *a priori* that the addressee of the message will behave accordingly—these signs function as behavioral directives. As a text genre, COVID-19 signs are unique in that, on the one hand, they have a very clear and strict informative content which is intended to prompt the addressee to obey and behave accordingly. Yet, on the other hand, some authors of the signs seek to maintain good relations with their addressees alongside informing them of practical guidelines. What linguistic means are used on the signs to reach this seemingly contradictory goal? One of the (likely unconscious) decisions that the author of a sign has to make is what to express explicitly and what to leave implicit. Using any markers of grammatical person on the sign is by no means compulsory or necessary. Thus, we regard the use of grammatical person as a meaningful choice by the sign author and set off from the broader ground to explore the linguistic expression of interpersonal relations on the signs.

In the common understanding of language as means for exchanging information, the other crucial function of language in communication is often overlooked: equally importantly, language creates and maintains social relations. The importance of this function of language has been brought to linguistics by B. Malinowski and referred to as phatic communion: “a type of speech in which ties of union are created by a mere exchange of words” (Malinowski, 1930 [1923], p. 315). We consider the COVID-19 sign as a genre of its own with special discourse roles, discursive moves and specific purposes (cf. Swales, 1990; Dancygier, 2021; Ogiermann and Bella, 2021). Drawing on Laver's further development of Malinowski's notion of “phatic communion” (see the next section for details), we relate the presence and absence of markers of 1st and 2nd grammatical person on the COVID-19 signs to solidarity and status relations between the authors of the signs and the addressees. Besides that, we investigate how the use of grammatical person is connected to other linguistic means of expressing authoritarity (cf. Svennevig, 2021)—the imperative mood and lexical expressions of authority, i.e., the nouns that refer to institutional authorities (the government, Health Board, etc.) and the legal regulations issued by them. As far as we know, there is no previous research that addresses these three linguistic means simultaneously in the context of public signs prompted by the crisis.

Initially, we qualitatively observed COVID-19 language in the case of Estonian door signs. As time went by, however, the pandemic produced a sufficient number of signs for quantitative analysis which became an important part of our study. As mentioned above, there are studies of the linguistic means used on public signs of the pre-COVID era, but they are mostly qualitative, i.e., analyzing the nature and variety of linguistic phenomena rather than the frequency or extent of it, due to the insufficient amount of data for a quantitative study. Despite the absence of such previous examples, there are already a few pioneering quantitative studies of the linguistic means used on COVID-19 signs (e.g., Ogiermann and Bella, 2021; Bella and Ogiermann, 2022).

The article is structured as follows: in the next section, the overview of the theoretical background is given; after that, data collection and organization are introduced, followed by the methodology used for the analysis. Then, in the section Analysis and Results, there are four subsections. The first three address analyzed linguistic phenomena: person, imperative mood, and expressions of authority, and the fourth introduces the interrelations among those. In the Discussion section, the results are considered in the context of previous research and the main conclusions of the analysis are presented. Finally, further research perspectives are discussed.

## Theoretical background

The term “phatic communion” was coined by Malinowski about 100 years ago to describe Trobriand Islanders' greeting formulae (Malinowski, 1930 [1923]). Malinowski's and our research share the concept of phatic communion: participants of the act of communication use specific linguistic means for social purposes—to create or maintain contact, to express solidarity, etc. Phatic communion (communication) has been studied and developed further by prominent linguists (e.g., Jakobson, 1960). Phatic communion has been said “to establish and maintain a feeling of social solidarity and well-being” (Lyons, 1968, p. 417). “[P]haticity may be best seen as a constellation of interactional goals that are potentially relevant to all contexts of human interchange” (Coupland et al., 1992, p. 211).

Our research draws from Laver who, in a further development of Malinowski's concept, divided phatic utterances into three tokens according to their orientation: (1) neutral (e.g., “Nice day”), (2) self-oriented (e.g., “My legs weren't made for these hills”), and (3) other-oriented (e.g., “Do you come here often?”). He associates the three categories (tokens) with the relative status of the parties in the communication situation: the use of language depends on whether one is in a lower or higher position than their partner, or their status is equal. If the social relations between the participants of a conversation are solidary, both personal (i.e., about oneself and the partner) and neutral (i.e., about something outside the participants, e.g., about the weather) phatic utterances are used in the conversation. In case the status of the parties is equal, but not solidary, neither the self-oriented nor the other-oriented categories are chosen, but only neutral utterances are used. If, however, there is a difference in social status between participants, the lower status participant (inferior) may use self-oriented phatic utterances, and the higher status participant (superior) may use the opposite strategy—other-oriented phatic utterances—in addition to neutral utterances that are available to speakers of any status (Laver, 1975, p. 223–224).

Phatic communion tokens (Laver, 1975, p. 223) are also in line with the previous studies about the grammatical category of person and social deixis (Siewierska, 2004, p. 214–215; cf. also Dancygier et al., in press). Grammatical person markers express the roles and relations of the participants of the act of communication: the speaker (first person), the addressee (second person), and a party talked about who is neither the speaker nor the addressee (third person) (Siewierska, 2004, p. 1). The connection between social relations and the use of the grammatical category of person has been researched before, e.g., in the use of personal pronouns. In many languages, 2nd person plural is used when addressing a person of higher status, and 2nd person singular is used when addressing a person of lower status (Brown and Gilman, 1960). The term “solidarity” has been used for the symmetric relationship (reciprocal use of 2nd person singular) between the speaker and addressee who have something in common, and contact between them should show like-mindedness (Brown and Gilman, 1960, p. 258). In the COVID-19 discourse, all of us as members of the global discourse community affected by the pandemic also shared a context of the situation (common ground of the pandemic), which made institutions and citizens somehow more equal and closer than in the pre-pandemic era.

Keeping that in mind, however, the study of language used on public signs is complicated by the fact that signs are one-way communication—we cannot account for the addressee's response to the received message. Therefore, we cannot compare the reciprocal use of pronouns or other linguistic means between the sign's author and the addressee. Laver's approach, on the other hand, allows us to interpret the establishment of social relations between the participants of a communication act based only on the choice of linguistic means by the initiator of the communication (the author of the sign). Thus, we found this approach to be a suitable tool for analyzing the status relations conveyed in the texts of the signs.

Previously, primarily lexical expressions have been researched as elements of phatic communication but that approach has also been used to explain the use of the vocative case (Jørgensen and Martinez, 2010) and emoticons (Aull, 2019). We decided to examine the usage of grammatical person as a means of expressing social relations on the COVID-19 signs because the author of a sign has no obligation to use 1st or 2nd person forms in the sign's text, as the message could as well be conveyed without them (e.g., *Wearing a mask is mandatory*). Thus, we want to explore how, by using and combining different grammatical forms of person, imperative mood, and expressions of authority, the author of a sign can thereby create different communication situations by expressing authority and solidarity [cf. also Dancygier et al. (in press) about emotional and interpersonal meanings on storefront signs in the time of COVID].

## Materials and methods

During the 1st wave of COVID-19 in Estonia (13.03.2020–15.10.2020), the only known and available method to stop the spread of the virus was reducing the contact between people to the bare minimum by social distancing. That included abruptly closing off most of the non-vital private and public services for an unknown period of time. However, the 2nd and 3rd wave (in Estonia 16.10.2020–25.08.2021 and 26.08.2021–29.11.2021, respectively) brought more elaborate restrictions, like mandatory mask-wearing, limitations to the number of people gathering and eventually the vaccination certificate, all meant to reduce the spread of the virus while preventing a full lockdown. During the second wave, the government even ran a campaign “Let's keep Estonia open!”, encouraging people to act responsibly and follow the restrictions.

**Figure 1:**
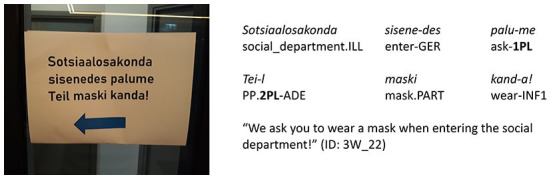
Estonian COVID-19 door sign. Linguistic means represented on the sign: type of person usage “both 1st and 2nd person” (expressions_of_authority_NO + imperative_NO).

The photos of the COVID-19 signs in our dataset were collected by the authors using crowdsourcing: an open call was distributed *via* social media and mailing lists instructing people to submit a photo of a COVID-19 sign (see Figure 1). In the call we asked contributors to submit the name of the institution or enterprise the sign was used at, the sign's location (e.g., door, floor, table, window, wall, cashier, elevator, elsewhere indoor), the date the photo was taken, and any other relevant information they wanted to share. Approximately a quarter of the signs were photographed by the authors of this article themselves. For the present study, we gathered 900 door signs (300 signs for each of the first three waves). Overall, 19,734 words (7298 + 6692 + 5744) were analyzed. We did our best to create the most varied possible assortment of signs by different type and size of commercial and non-commercial institutions. Most of the signs were created by establishments themselves, even though there were printable posters compiled by the governmental institutions available on the official webpage of the crisis communication. Our data includes signs about closing and reorganizing businesses, canceling events, keeping social distance, disinfecting hands, limiting the number of people in an area, wearing a mask, presenting a certificate, etc. The sample includes signs from large businesses (e.g., chain stores like Rimi and Maxima), small businesses (e.g., local restaurants, pubs, coffee shops, beauty salons) and non-business places (e.g., educational institutions, hospitals, libraries, museums, theaters, churches). Duplicates were excluded from our sample (e.g., the signs that were used in many stores of large chains were included in the sample once). The geographical distribution of the signs was approximately following: ½ from Tartu, ¼ from Tallinn, and ¼ from the rest of Estonia.

The door sign was chosen for the analysis as the most common COVID-19 sign since it marks the border between the sign author's space and the addressee's space and could thus be described as a barrier between the sign's author and the addressee (cf. Dancygier et al., in press). Door signs were also relevant because different measures applied in outdoor and indoor spaces. Thus, the signs marked a border between different regulations or starting points of the regulations. Signs on the windows or notice boards next to the entrances were also considered door signs. Example signs in this article were deliberately chosen about wearing a mask to enable the reader to compare the use of linguistic means of content as homogenous as possible.

**Figure 2:**
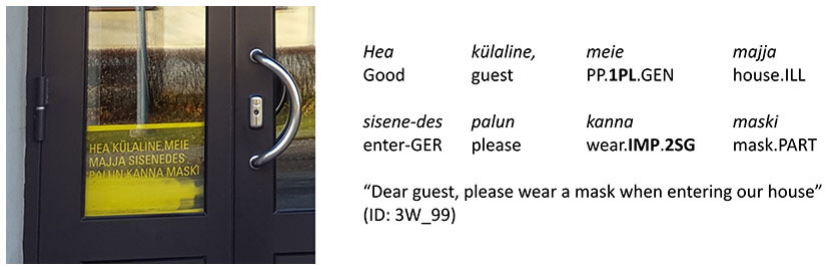
Type of person usage “both 1st and 2nd person” (expressions_of_authority_NO + imperative_YES).

As linguists, we were interested in the linguistic means through which the authority and solidarity in the message were conveyed. Extra-language modalities (colors used on the sign, the size and shape of the font, company logos, text placement on the sign, etc.) were left out of this study even though we acknowledge that these also play a significant role. Similarly, we have excluded from our analysis the other phatic linguistic means that can be found on the signs, e.g., greetings (e.g., *hea külaline* “dear guest”, see Figure 2) and other linguistic expressions of politeness (e.g., *palun* “please”, see Figure 2, *aitäh* “thanks”, see **Figure 4**). Ogiermann and Bella (2021) analyzed such expressive speech acts on the COVID-19 closure signs in London and Athens and found that they are much more frequent on COVID-19 signs than on other closure signs. On COVID-19 signs they do not function so much as formal expressions of politeness but often rather as means of creating and maintaining emotional relationships, i.e., fulfilling a phatic function similar to what we assumed of the forms of grammatical person. Thus, it would be reasonable to include expressive speech acts in the analysis of grammatical person in the future. We have not done it yet because it requires a time-consuming manual coding, while the grammatical person is accessible through tools of automatic language analysis.

Even though multilingualism of the signs has been a traditional topic in the Linguistic Landscape paradigm, and although we did have multilingual signs in our sample (163 multilingual signs out of 900: Estonian—English 84, Estonian—Russian 53, and Estonian—English—Russian 26), we analyzed only Estonian texts on the signs and presented English translations of Estonian texts in examples.

The text and metadata of the signs were organized in an Excel table. Texts were automatically analyzed (identifying expressions of authority, 1st and 2nd grammatical person, and mood) using the Python package ESTNLTK (Orasmaa et al., 2016). For statistical analysis, we used χ^2^-tests to determine whether significant relationships existed between the studied linguistic features. The statistically significant results are reported. Statistical analysis was performed using the chisq.test() function of the R software package “stats” version 4.0.5 (R Core Team, 2021).

## Analysis and results

In this section, linguistic phenomena on the COVID-19 signs—grammatical person, imperative mood, and expressions of authority in Estonian—are introduced. Then, the quantitative analysis of the presence and absence of imperative mood and expressions of authority is presented in relation to types of person usage. Finally, most frequent combinations of the type of the person usage, imperative mood and lexical expressions of authority are described, providing the basis for further discussion about expressing authority and solidarity on a sign.

### Person

In Estonian, grammatical person is expressed by personal pronouns (PP) and verbal suffixes, which are combinations of person (1, 2, 3) and number - singular (SG) and plural (PL).^1^ In the imperative mood, verb forms are usually used without personal pronouns (e.g., *Kand-ke maski*! “Wear-IMP.2PL a mask!”). In the indicative mood, verbal suffixes are sometimes used simultaneously with personal pronouns in subject function (e.g., ***me*** *kanna*-***me*** “PP.1PL wear-1PL”). Still, in indicative affirmative, it is also quite common to omit (pro-drop) personal pronouns (e.g., *kanna*-**me** “wear-1PL”) as the person is marked in the verb (-**me** “1PL”). However, since there are no explicit person markers in indicative negative, a pronoun is obligatory in the case of negation (e.g., ***me*** *ei kanna* “PP.1PL NEG wear.CONNEG”). In all syntactic functions other than subject, the personal pronoun is not omitted because the verb form expresses only the grammatical person of the subject (see Figure 1).

Table 1 presents all forms of the grammatical person which were included in the analysis. From the verbal paradigm, affirmative and negative imperative and affirmative indicative present tense forms of 1st and 2nd person singular and plural were included. Negative forms of the indicative mood were excluded from the analysis because verbal negation does not explicitly express grammatical person in Estonian. As of personal pronouns, 1st and 2nd person singular and plural^2^ in both long (e.g., *meie* “we”) and short forms (e.g., *me* “we”) were included. Additionally, there are 14 cases in Estonian^3^, all of which can be applied to all personal pronouns, including the genitive form which also functions as a possessive pronoun like *my* or *our* in English (see Figure 2). All the forms of 1st and 2nd person pronouns in all cases were included in the automatic analysis [For more detailed description of the person markers in Estonian, see Erelt, 2003, p. 53 (about verbal markers) and Pool, 1999 (pronominal markers).].

**Table 1:** The grammatical person forms of Estonian personal pronouns and verbs taken into account in the present study (in the imperative mood, only 1PL has the person marker; marker -*ge*- in 1PL and 2PL forms after the stem *püsi*- of the example verb *püsima* “to stay” indicates imperative mood).

**Grammatical person**	**Pronouns**	**Verbs**		
		**Indicative**	**Imperative**	**Imperative**
		**affirmative**	**affirmative**	**negative**
1SG	***mina**/**ma***	*püsi-**n***	-	-
1PL	***meie**/**me***	*püsi-**me***	*püsi-ge-**m***	ä*r-ge-**m** püsi-ge-**m***
2SG	***sina**/**sa***	*püsi-**d***	*püsi-Ø*	ä*ra-Ø püsi-Ø*
2PL	***teie**/**te***	*püsi-**te***	*püsi-ge*	ä*r-ge püsi-ge*

**Figure 3:**
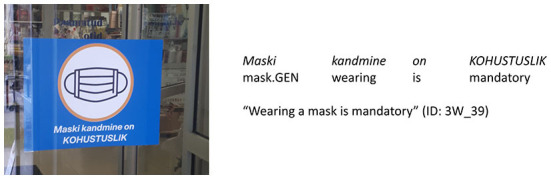
Type of person usage “neither 1st nor 2nd person” (expressions_of_authority_NO + imperative_NO).

**Figure 4:**
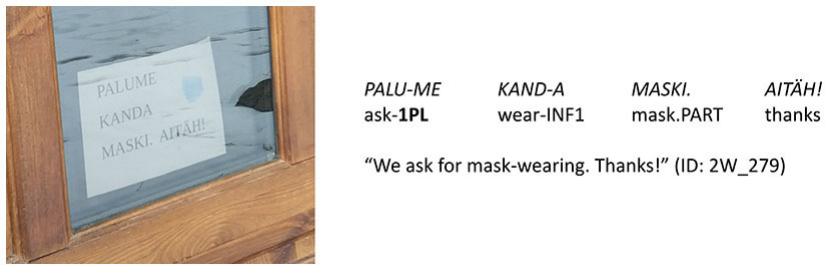
Type of person usage “1st person only” (expressions_of_authority_NO + imperative_NO).

**Figure 5:**
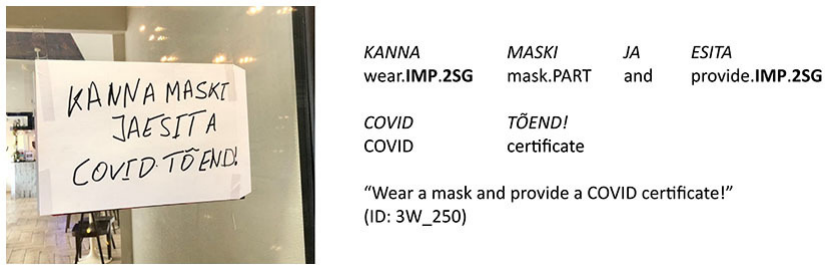
Type of person usage “2nd person only” (expressions_of_authority_NO + imperative_YES).

**Figure 6:**
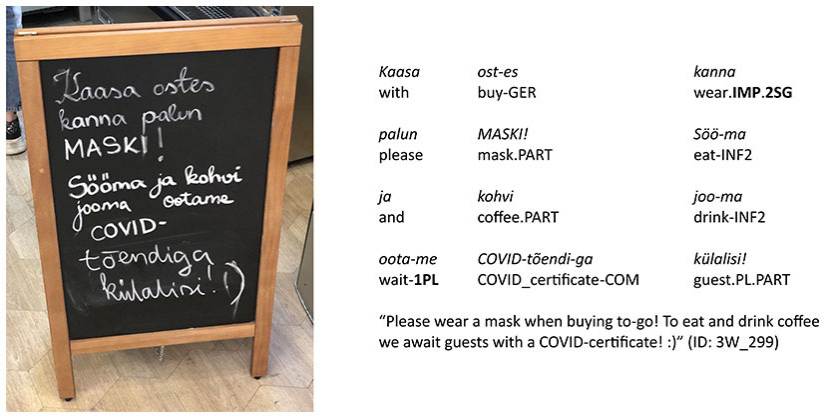
Type of person usage “both 1st and 2nd person” (expressions_of_authority_NO + imperative_YES).

We used automatic search for the person markers on all signs and distinguished four types of person usage found in details in Table 2: (1) neither 1st nor 2nd person (see Figure 3); (2) 1st person only (see Figure 4); (3) 2nd person only (see Figure 5); (4) both 1st and 2nd person (see Figure 6).

**Table 2:** Types of the person usage on the Estonian COVID-19 signs (V, verb; PP, personal pronoun; SG, singular; PL, plural; 1,2, person).

**Type of the**	**1st person forms of**	**2nd person forms of**	**Signs**
**person usage**	**which at least one is**	**which at least one is**	**(*n* = 900)**
	**present on the sign: V_1SG**,	**present on the sign: V_2SG**,
	**V_1PL, PP_1SG, PP_1PL**	**V_2PL, PP_2SG, PP_2PL**	
Neither 1st nor 2nd person	No	No	201
1st person only	Yes	No	237
2nd person only	No	Yes	215
Both 1st and 2nd person	Yes	Yes	247

### Imperative mood

The imperative mood in Estonian has means to express affirmative and negative polarity, person, and number. First person singular form of the imperative is absent (as it is illogical to give orders to oneself) and 2nd person singular is unmarked. In the plural forms of 1st and 2nd person, the imperative marker *ge/ke* is used. For more detailed description of the imperative in Estonian, see Metslang (2004), Metslang and Sepper (2010, p. 533–537). The overview of the imperative forms included in this study is given in Table 1 above.

**Figure 7:**
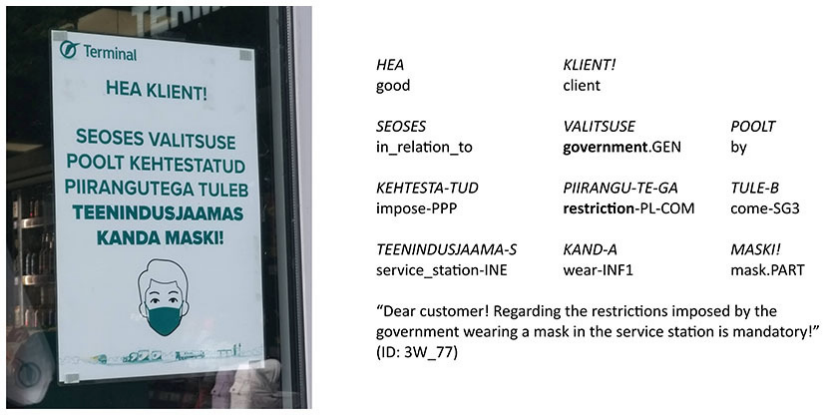
Type of person usage “neither 1st nor 2nd person” (expressions_of_authority_YES + imperative_NO).

The imperative is one of the most common linguistic means to express status relations. Usually, only higher-status participants are eligible to give orders to the lower-status participants in the act of communication. The main functions of imperative mood are to deliver requests, orders, commands, and demands, and it also calls for the addressee's responsibility. Other-oriented 2nd person forms are the central elements of the imperative mood paradigm. These forms imply that the speaker does not submit to the action referred to by the behavioral directive: the sender of the message is the source of the command, and the addressee is the performer of the commanded action. In the COVID-19 discourse, sign authors were in the specific discourse role of communicating the message, initially delivered by the government, to the addressees who are expected to behave in a way the sign instructs (e.g., wear a mask). Using the imperative, the author of the sign directly presents themselves as the author of the behavioral directive. When the imperative is not used, the original author of the order (e.g., the government) is often referred to explicitly with an expression of authority (see Figure 7).

There is only one person and number form in the Estonian imperative mood paradigm that includes the speaker−1st person plural *kand-ke-m* “let's wear”*, hoid-ke-m* “let's keep”*, püsi-ge-m* “let's stay”. Because it indicates the speaker as well as the addressee, it is both self- and other-oriented. This form appeared on 13 signs (5 times in the type “only 1st person” and 8 times in the type “both 1st and 2nd person”), which is not much but still remarkable since this form has been considered very rare, used only in the high style so far (Metslang and Sepper, 2010, p. 534). All other uses of person in the imperative are other-oriented.

An automatic search of markers of the imperative mood was conducted, and as a result, two groups of the signs were formed depending on whether the sign included at least one instance of imperative mood or not. The imperative was present on 401 (45%) signs (see Figures 1, 3, 4) and not present on 499 (55%) signs (see Figures 2, 5, 6).

### Expressions of authority

We presumed that referring to legal measures issued by governmental institutions would also be a strategy sign authors use to achieve an expected behavior by the addressees (cf. Svennevig, 2021; Bella and Ogiermann, 2022). In addition to their primary function of supporting authority of the message, those expressions are used to establish a discourse community: the author of the sign assumes that the reference is accessible and understandable to the addressee. To find out how and when this intertextual reference is used, expressions referring to institutional authorities and legal acts were manually extracted from our dataset and converted into the following keywords: *eriolukord* “state of emergency”, *vabariik* “republic”, *riik* “state”, *valitsus* “government”, *terviseamet* “Health Board”, *korraldus* “order”, *otsus* “decision”, *nõue* “demand”, *piirang* “restriction”, *meede* “means”, *määrus* “decree”, *ettekirjutus* “guideline”, *juhis* “instruction”, *sisekorraeeskiri* “internal rules” (cf. also Tragel and Tomson, 2022).

Next, the automatic search of keyword lemmas was conducted on the condition that at least one of these expressions would be present on the sign. The search resulted in two groups: signs with (in total 231 of 900 signs, i.e., 26%; see Figure 7) and without expression of authority (669 of 900 signs, i.e., 74%; see Figures 1–6).

### Interrelations between the types of person usage, imperative mood, and expressions of authority

In Table 3, the distribution of the presence and absence of the imperative mood and expressions of authority is presented within each type of person usage. This table enables us to simultaneously follow the correlations between the three linguistic features we attribute to authority and solidarity dynamics: use of person, expressions of authority and imperative mood. Values of the four types of person usage are (1) neither 1st nor 2nd person; (2) 1st person only; (3) 2nd person only, and (4) both 1st and 2nd person. Features “expression of authority” and “imperative” have two values: (1) yes (present) and (2) no (absent).

**Table 3:** The distribution of the presence (YES) and absence (NO) of the imperative mood and expressions of authority within each type of person usage.

**Type of person usage**	**Expressions of authority**	**Imperative**		
		**YES**	**NO**	**TOTAL**
Neither 1st nor 2nd person	YES	0%	27%	27%
(*n* = 201)	NO	0%	73%	73%
	TOTAL	0%	100%	100%
1st person only	YES	1%	29%	30%
(*n* = 237)	NO	1%	69%	70%
	TOTAL	2%	98%	100%
2nd person only	YES	9%	1%	10%
(*n* = 215)	NO	84%	6%	90%
	TOTAL	93%	7%	100%
Both 1st and 2nd person	YES	26%	9%	35%
(*n* = 247)	NO	53%	12%	65%
	TOTAL	79%	21%	100%

In the table, the darker cells with the same color represent higher values and the lighter cells represent lower values. The red cells in the last column of each type of person usage show the distribution of signs with and without expressions of authority. The blue cells in the last row of every type of person usage show the distribution of signs with and without the imperative mood. The purple cells show the distribution of signs between the two features simultaneously: the presence or absence of expressions of authority and the imperative mood.

#### Expressions of authority and types of person usage

Table 3 shows that the number of signs without expressions of authority is larger than the number of signs with them in all types of person usage. The percentages, however, vary: the type “2nd person only” has significantly fewer signs with expressions of authority (10%), while the type “both 1st and 2nd person” had the most (35%).

**Figure 8:**
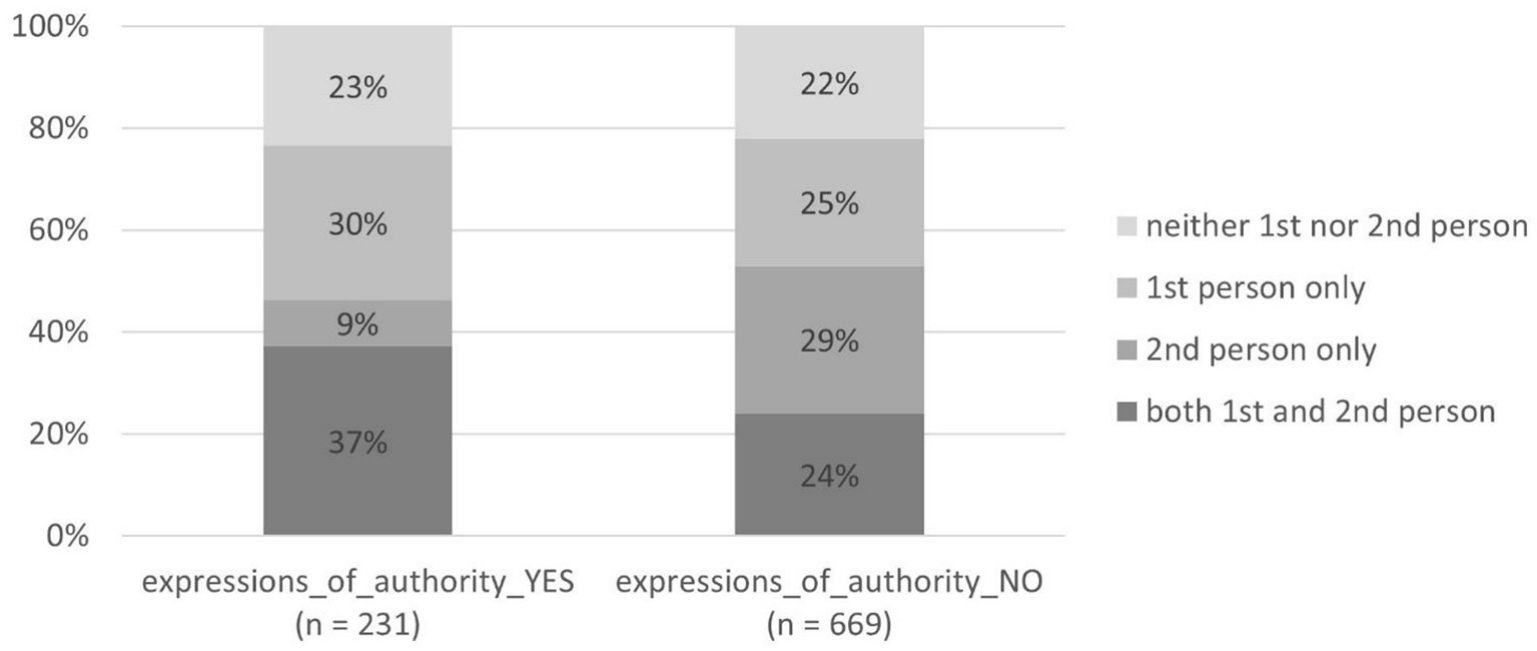
Expressions of authority and types of person usage.

The difference between the use of person markers on signs with or without expressions of authority is also statistically significant [$\chi_{\left(3\right)}^{2}$ = 41.34, *p* < 0.0001]: it can be seen that expressions of authority and the type “2nd person only” tend not to be used together on one sign (see Figure 8).

#### Imperative mood and 2nd person with and without 1st person

**Figure 9:**
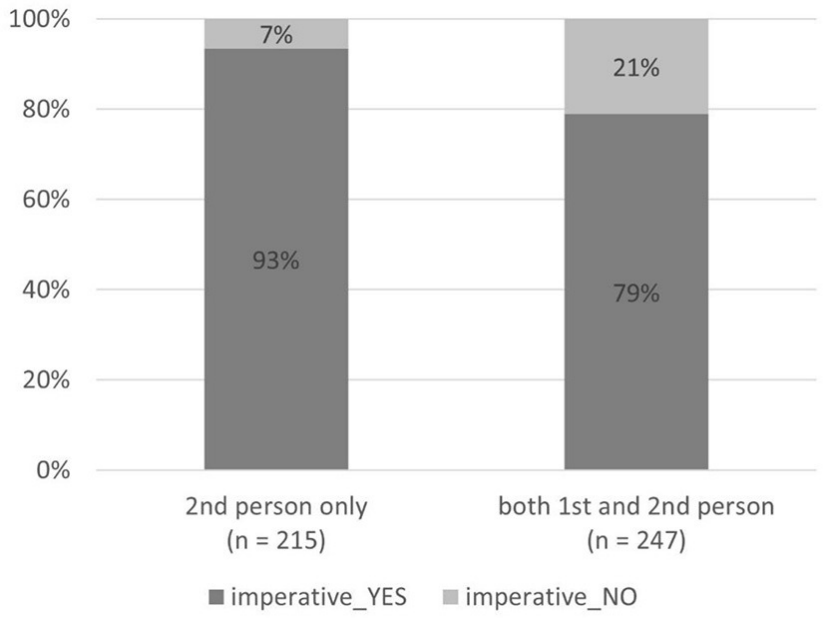
Imperative mood and 2nd person with and without 1st person.

As for the imperative mood, it cannot be used in the type “neither 1st nor 2nd person”, and it is very rare in the type “1st person only” (5 times in this type, see the section about imperative mood above). The imperative mood is used on most signs of the type “2nd person only” (93% of the signs have the imperative mood, 7% do not, see Figure 9). On the signs where 1st person is used in addition to 2nd person (i.e., the type “both 1st and 2nd person”), the imperative mood is used much less (79% of the signs have the imperative mood, 21% do not), although the presence of 2nd person would allow using the imperative mood in this type just as often as on the signs of the type “2nd person only”. The difference in using the imperative mood in these two types of person usage is statistically significant [$\chi_{\left(1\right)}^{2}$ = 18.7, *p* < 0.0001].

#### Imperative mood and expressions of authority

**Figure 10:**
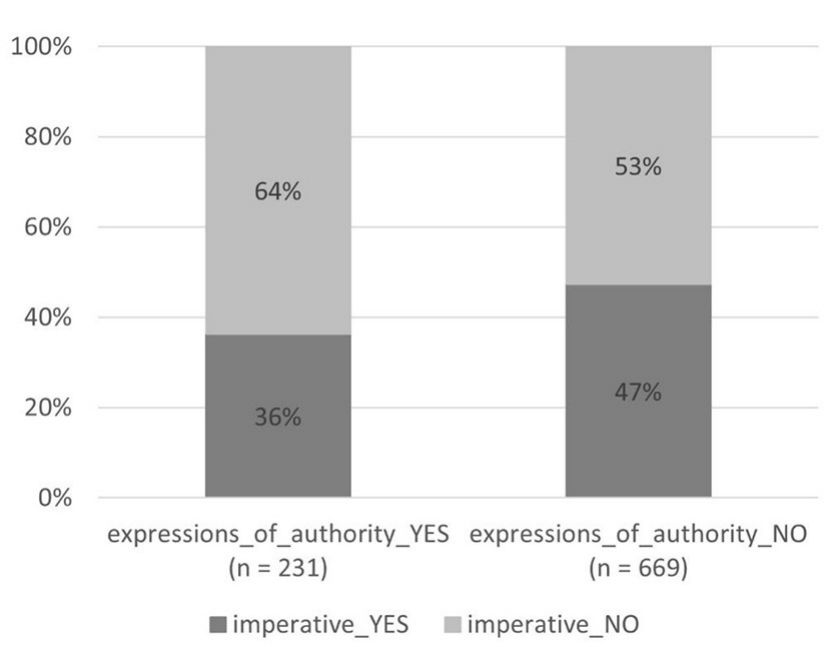
Imperative mood and expressions of authority (four types of person usage together).

The statistical analysis of all four types of person usage together shows also a weak negative correlation between the features “expressions of authority” and “imperative mood” [$\chi_{\left(1\right)}^{2}$ = 8.0, *p* = 0.005]: the signs with expressions of authority have less imperative forms (84 signs of 231, i.e., 36%) than the signs without expressions of authority (317 signs of 669, i.e., 47%)—see Figure 10. Hence, it is not very common to use the imperative mood and expressions of authority—the two means of implementing authority—together. The manifestation of their combination in each type of person usage is analyzed in the next subsection.

#### Most frequent combinations of the type of the person usage, imperative mood and expressions of authority

**Figure 11:**
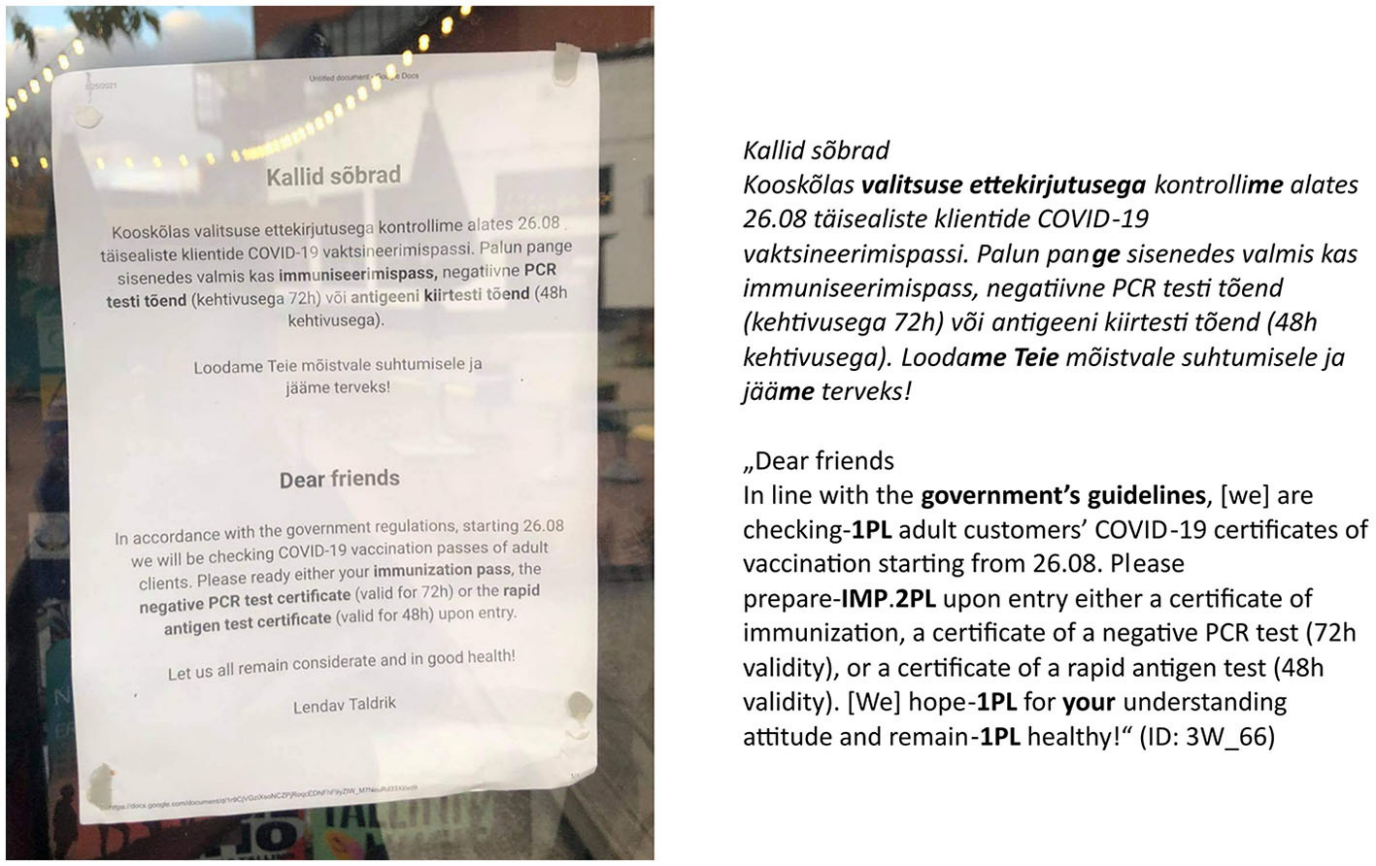
Type of person usage “both 1st and 2nd person” (expressions_of_authority_YES + imperative_YES).

**Figure 12:**
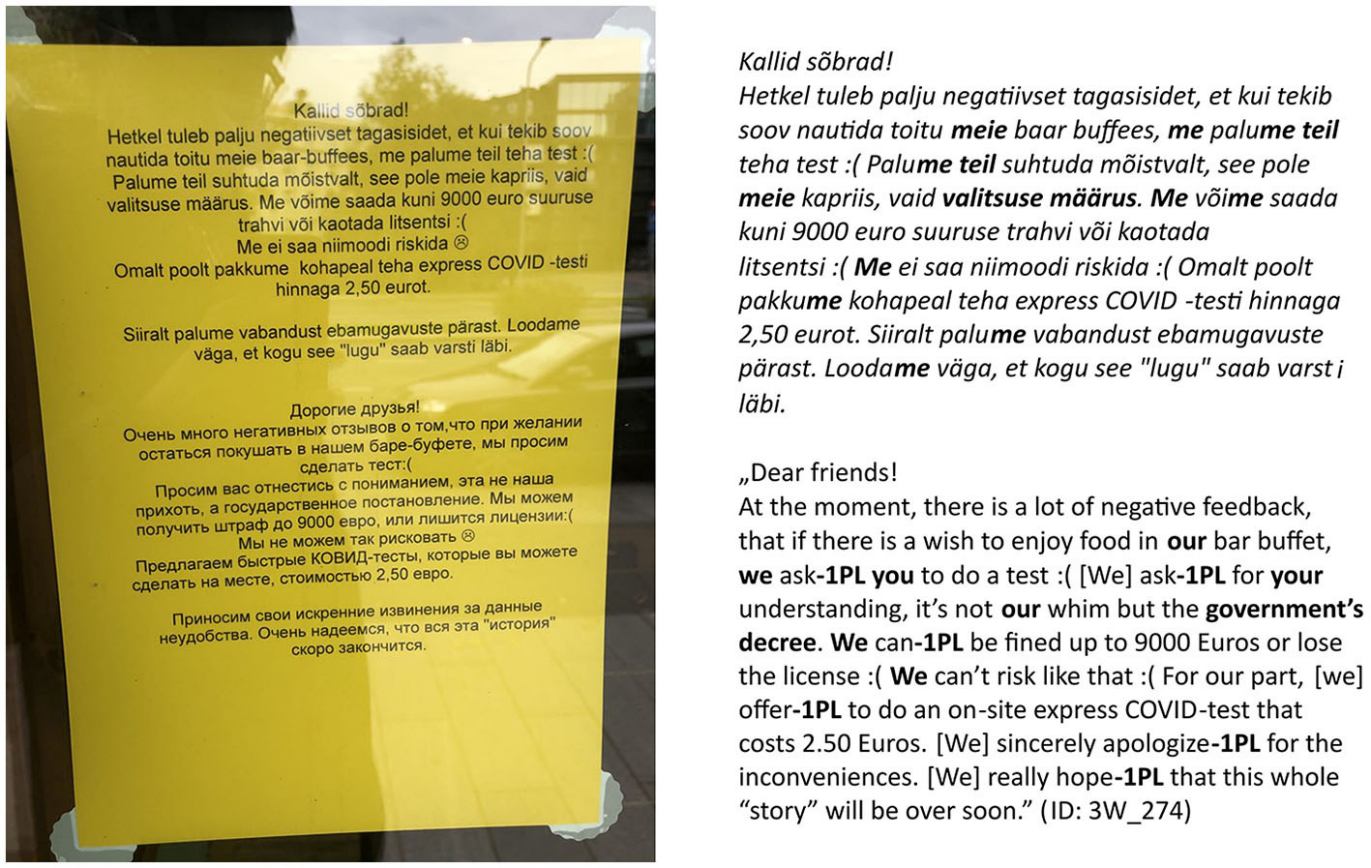
Type of person usage “both 1st and 2nd person” (expressions_of_authority_YES + imperative_NO).

The most frequent combinations of the type of person usage, imperative mood, and expressions of authority can be found in the darkest purple cells in Table 3 above:

1. of the 201 signs of the type “neither 1st nor 2nd person”, the combination of “expressions_of_authority_NO + imperative_NO” is the most frequent (147 signs, i.e., 73%; see Figure 3), but the combination “expressions_of_authority_YES + imperative_NO” is also rather frequent (54 signs, i.e., 27%);
2. of the 237 signs of the type “1st person only”, the combination of “expressions_of_authority_NO + imperative_NO” is the most frequent (164 signs, i.e., 69%; see Figure 4), but the combination “expressions_of_authority_YES + imperative_NO” is also rather frequent (68 signs, i.e., 29%);
3. of the 215 signs of the type “2nd person only”, the combination of “expressions_of_authority_NO + imperative_YES” is the most frequent (182 signs, i.e., 84%; see Figure 5);
4. of the 247 signs of the type “both 1st and 2nd person”, the most frequent combinations are “expressions_of_authority_NO + imperative_YES” (132 signs, i.e., 53%; see Figure 6) and “expressions_of_authority_YES + imperative_YES” (63 signs, i.e., 26%; see Figure 11). Additionally, the other two combinations were more present here than the less frequent combinations in the other types of person usage. The combination “expressions_of_authority_YES + imperative_NO” (23 signs, i.e., 9 %) is represented in Figure 12 and the combination “expressions_of_authority_NO + imperative_NO” (29 signs, i.e., 12%) is represented in Figure 1.

For a native speaker of Estonian and a member of local COVID-19 discourse community, the absence of the imperative mood (Figures 1, 9) makes the signs sound less authoritative than the signs with the imperative mood present (Figures 6, 8). However, it does create a feeling of solidarity through the use of both 1st and 2nd person. Furthermore, the use of expressions of authority referring to an entity of higher status than both the sign's author and the addressee (see Figure 9) is likely to create a higher willingness in the addressee to collaborate than using only 2nd person imperative on the sign (see Figure 5), which leaves the addressee at an inferior position. In the following discussion, we will look deeper into expressing and combining authority and solidarity. However, the scientific verification of this argumentation requires a sign-processing experiment we are currently designing for further research.

## Discussion

In the previous section, we analyzed interrelations of the types of person usage, the imperative mood, and expressions of authority. We based our research on Malinowski's notion of “phatic communion” (Malinowski, 1930 [1923]) as a contact creation device. Relying on Laver's notions of “self-oriented” and “other-oriented” (Laver, 1975), we regarded personal pronouns and verb markers of 1st person as self-oriented and of 2nd person as other-oriented means of language. We found that using or not using 1st person and/or 2nd person forms on the sign establishes communication situations that express different authority and solidarity relations between the sign's author and the addressee.

Most significant findings about the interrelations of expressions of authority, imperative mood, and use of person markers could be concluded as follows:

–On the signs with only other-oriented 2nd person (without 1st person) lexical expressions of authority are infrequent and authority is often expressed by imperative mood;
–On the signs with both self-oriented 1st person and other-oriented 2nd person, however, imperative mood is used less often. In this case, what add authority to the message are lexical expressions of authority which are used much more frequently on the signs in this type than on the signs of the type “2nd person only”.

In everyday conversations, using the imperative mood in 2nd person is a natural way to address the interlocutor. However, using the other-oriented 2nd person (either with or without 1st person) also seems to connote the participants' status relations in the act of communication. It seems that using only 2nd person (i.e., saying something about the addressee) is more authoritative than using it along with 1st person (i.e., saying something about oneself as the author). The imperative mood—which can be assumed to “empower” the sign's message—is used more often on the signs of the type “2nd person only”. The imperative mood (with the exception of the 1PL form) expresses non-solidary relations between participants: the author of the sign finds himself eligible to ask the addressee to behave in a certain way, consequently placing himself in the superior position in the communication situation.

Furthermore, why are expressions of authority used significantly more often on the signs of the type “both 1st and 2nd person” than on the signs of the type “2nd person only”? A possible explanation is that it has to do with establishing and maintaining the solidarity relationship between the participants of the communication act: expression of authority would be used to justify behavioral directives or other pieces of potentially unpleasant information that must be communicated to the addressee. On the other hand, referring to oneself (i.e., using 1st person) reduces the authoritarianism of the sign's message by signaling the equality of the participants instead of the author's authority—the author of the sign and the addressee are both subjected to a superior authority to which the author invites the addressee to submit. The wide use of the lexical expressions of authority at the beginning of the pandemic justifies itself as people were adapting to an unfamiliar situation, for which restrictions needed explanation and justification. However, after some time, the addressee of a sign as a member of the global and local COVID-19 discourse could be assumed to know and understand the situation. Hence, such expressions were more likely to be redundant on the signs, but they did not disappear from them. This phenomenon presents itself as a general feature of the COVID-19 discourse, where community members possess shared knowledge about the situation but still decide to explicitly express it to emphasize joint responsibility as a means of coping with the crisis.

As for the remaining two types of person usage, signs of the type “1st person only” are associated with solidarity rather than authority, although lexical expressions of authority (if present on the sign) make them more authoritative. On the contrary, the signs of the type “neither 1st nor 2nd person” create no solidarity in the addressee. Instead, these signs convey the message without creating personal contact with the addressee (cf. Laver, 1975, neutral type).

Although the elements with phatic function can be very small, their role in communication is huge (McCarthy, 2003, p. 60). This also applies to the pronouns and verb markers analyzed in this article. The easiest way of presenting a behavioral directive is, surely, using the imperative forms of 2nd person, which might be the case when the sign's author is already tired of the prolonged situation and may want it to be over quickly. However, the addressee might also be tired of living in a world full of restrictions and might not be bothered to make an effort anymore. For a tired addressee, it is especially important to turn more attention to the language of a message (Barron, 2012, p. 71). When an already long crisis escalates even further, finding the right language for conveying instructions might become even more important.

It was established already in the pre-COVID era that the more interested a sign's author is in maintaining good relations with their customer (i.e., the addressee), the more solidarity they try to show in their choice of words, especially when following the guidelines is not legally mandatory but dependent on the customer's goodwill (Svennevig, 2021, p. 182). Even when the guidelines on the COVID-19 signs were legally mandatory, the authors of the signs still used additional linguistic means to create solidarity. Small-business entrepreneurs were thereat probably the most invested in creating the signs during the COVID-19 crisis since their income depended on maintaining good customer relations. Although, on the one hand, this might seem motivated by self-interest, on the other hand, making the addressee feel equality and solidarity is an integral part of crisis communication. It could be seen that private entrepreneurs served as a good example of how an addressee could be reached in a difficult situation. Thus, researching the signs of the COVID-19 crisis can also give us a broader awareness of the use of language in crisis communication, e.g., how to avoid putting the addressee of legally mandatory guidelines in the position of an inferior following orders and instead express their being a member of the society who does their part in coping with the difficult situation and submits to the sign communication effectiveness.

Messages where the author communicates empathy and hopes for empathy on the addressee's part are the ones that call for cooperation and shared effort the most. The importance of expressing solidarity and friendship in the communication between the authors of the COVID-19 signs (business enterprises) and the addressees (customers) has also been addressed by Dancygier et al. (in press). According to our analysis, a distinctive feature of such messages is using both 1st and 2nd grammatical person on the sign. Authority can be added by using lexical expression of authority, i.e., making an intertextual reference to governmental institutions to which both the author and the addressee of the sign are equally submitted. Even the imperative mood, which usually expresses authority, does not emphasize the gap between the statuses of the parties when used together with both 1st and 2nd person, compared to signs where only 2nd person is used.

Additionally, psychologists have also revealed that the best strategy for opening a conflict discussion is to use statements that include I-language (instead of you-language) and communicate “both self- and other-perspective”, because such use of language significantly reduces “perceptions of hostility” (Rogers et al., 2018). The research of psychologists focuses on the message's content and not the grammatical means of language (e.g., if you-language statements include only the pronoun *you*, then I-language statements can include both the pronouns *I* and *you*, and not necessarily just *I*), just like Laver (1975), whom we are drawing on, did not directly associate self-oriented and other-oriented tokens with grammatical elements. However, a quantitative analysis of our empirical data, which demonstrates that since the use of grammatical person (self-oriented 1st person and other-oriented 2nd person) on COVID-19 signs is systematically connected to other linguistic means of expressing authority (the imperative mood and lexical expressions of authority), confirms Laver's claim that the use of self-oriented and other-oriented utterances reflects the status relations of the interlocuters. Signs are a one-way communication act, thus the use of language on the signs expresses the role which the author of the sign takes for themselves in relation to the addressee—whether they present themselves as an authority to whom the addressee must submit, or as an equal partner, who invites the reader to together submit to some kind of external authority. In their research of the COVID-19 signs in UK and Greece, Bella and Ogiermann interpreted this kind of role-taking as a creation of identity by the authors of the signs and found that “[a]mong these identities, the one of the self-directed social actor turns to be most crucial” (Bella and Ogiermann, 2022, p. 644).

Thus, not only is what we tell each other important but also how we do it. We create and maintain social relations by using certain linguistic means because different means create different communication situations and evoke different feelings among the parties, influencing their behavior.

## Ideas for further research

The study of COVID-19 signage is a multifaceted, multidisciplinary area of which we have only scratched the surface in our article. Many other questions remain to be studied to gain further insight into how solidarity and authority are expressed on the signs. An important comparison excluded from this article was that of institutions (e.g., grocery stores, pharmacies, restaurants, etc.) which we plan to research in the future. We also have not yet looked into the multilingualism of the signs, a traditional topic in the framework of the Linguistic Landscape, due to multilingual signs not being very widespread in Estonia, apart from the multilingual community in the capital Tallinn.

There are quite a few signs without imperative forms and expressions of authority in our data, e.g., in the type “neither 1st nor 2nd person” but also in other types of person usage. How does the author of the sign achieve authority in these acts of communication? We presume other means, which we did not analyze in this study, have been used instead, e.g., modal verbs and other modal expressions that have been mentioned in previous studies of signs (e.g., Svennevig, 2021), e.g., *needs to be, must be, can be, is allowed, is mandatory, is needed*. The visual aspect could also be used to instill authority (e.g., the company's logo, colors, capital letters, etc.). In the future, it would also be reasonable to investigate these factors in relation to the use of person, self- and other-oriented language, and the expression of authority and solidarity.

Further future directions are also the politeness distinction in 2nd person singular and plural (the connections between politeness, formality, and solidarity in 2PL) as well as other expressions of politeness (e.g., *please, thank you, we thank you, we excuse*, etc.)—do these expressions add to the solidarity conveyed by the message? Other possible topics for further research include the distribution of verb forms and personal pronouns in different types of person usage, the distinction between inclusive and exclusive 1PL, the placement of the expression of authority in the text, etc. Investigating the use of negation would also provide valuable insight into the research (e.g., *We do not offer service without a mask* is highly unlikely to create any sense of solidarity).

As other potential means of solidarity, handwritten signs can be researched for their ability to create more intimate contact between the author and the addressee (see Hua, 2021); likewise, the greetings at the margins of the signs (see Ogiermann and Bella, 2021) for which a “self-oriented” and “other-oriented” analysis could be applied (cf. Laver, 1981) as well as an analysis on how the author of the sign refers to themselves at the end of the sign. Dancygier et al. (in press) distinguish two ways the author of a COVID-19 sign relates to the addressee: (1) a compliant addressee or (2) a partner in a friendly exchange. How the division of these roles—authoritative commands or expressions of friendship as well as other markers of social deixis—are expressed in Estonian can, in the future, be researched based on our data. Furthermore, signs' formality and informality could be analyzed automatically by a resource currently in development, using genre-independent methodology for analyzing Estonian texts (Gailit, 2021; cf. Sheikha and Inkpen, 2012).

Lastly, it would be interesting to compare the signs in different countries and languages, e.g., according to the cultural scripts' approach (e.g., Wierzbicka, 1998), and see how different cultures create a sense of solidarity and achieve effective communication through public signs.

## Data availability statement

The original contributions presented in the study are included in the article/supplementary material, further inquiries can be directed to the corresponding author/s.

## Author contributions

Both authors listed have made a substantial, direct, and intellectual contribution to the work and approved it for publication.

## Footnotes

^1^Abbreviations for grammatical categories used in the examples in this article: (-) hyphen, is used to separate segmentable morphemes; (.) period, is used to separate non-segmentable morphemes; 1, 1st person; 2, 2nd person; 3, 3rd person; ADE, adessive case; COM, comitative case; CONNEG, connegative; GEN, genitive case; GER, gerund; ILL, illative case; IMP, imperative; INE, inessive case; INF1, da-infinitive; INF2, ma-infinitive; NEG, negation; PART, partitive case; PL, plural; PP, personal pronoun; PPP, past passive participle; SG, singular; V, verb.

^2^In Estonian, there are no grammatical means for distinguishing exclusive and inclusive 1PL.

^3^In Table 1, nominative case of the personal pronoun is presented.

## References

[B1] Aull B. (2019). A study of phatic emoji use in whatsapp communication. Internet Pragmat. 2, 206–232. 10.1075/ip.00029.aul

[B2] Barron A. (2012). Public Information Messages: A Contrastive Genre Analysis of State-Citizen Communication. Amsterdam: John Benjamins. 10.1075/pbns.222

[B3] Bella S. Ogiermann E. (2022). Accounts as acts of identity. Justifying business closures on COVID-19 public signs in Athens and London. Pragmatics 32, 620–647. 10.1075/prag.21033.bel

[B4] Blommaert J. (2013). Ethnography, Superdiversity and Linguistic Landscapes: Chronicles of Complexity. Bristol, Buffalo: Multilingual Matters. 10.21832/9781783090419

[B5] Bonner M. (2016). Formulierungsmuster auf dänischen Schildern. Folia Scand. 20, 15–39. 10.1515/fsp-2016-0023

[B6] Brown R. Gilman A. (1960). The pronouns of authority and solidarity, in Style in Language, ed Sebeok T. A. (Cambridge: MIT Press) 253–276.

[B7] Coupland J. Coupland N. Robinson J. D. (1992). 'How are you?': negotiating phatic communion. Lang. Soc. 21, 207–230. 10.1017/S0047404500015268

[B8] Dancygier B. (2021). Fictive deixis, direct discourse, and viewpoint networks. Front. Commun. 6, 624334. 10.3389/fcomm.2021.624334

[B9] Dancygier B. Lee D. Lou A. Wong K. (in press). Standing together by standing apart: distance, safety, fictive deixis in the COVID-19 storefront communication, in COVID 19: Metaphor Metonymy across Languages Cultures, eds Wen X. Lu W-L. Kövecses Z. (Amsterdam: John Benjamins Publishing Company).

[B10] Doroja-Cadiente G. Valdez P. N. (2019). A linguistic landscape analysis of public signs after Typhoon Haiyan. Int. J. Asia Pacifc Stud. 15, 33–57. 10.21315/ijaps2019.15.1.2

[B11] Erelt M. (ed.). (2003). Estonian Language. Tallinn: Estonian Academy Publishers.

[B12] Ferenčík M. (2018). Im/politeness on the move: a study of regulatory discourse practices in Slovakia's centre of tourism. J. Pragmat. 134, 183–198. 10.1016/j.pragma.2018.05.011

[B13] Gailit K. G. (2021). Spontaansuse ja formaalsuse kui dimensionaalse tekstimudeli dimensioonide automaatne hindamine veebitekstides / Evaluating the spontaneity and formality of online texts as dimensions of the dimensional text model. [bachelor thesis]. University of Tartu, Tartu, Estonia.

[B14] Hua Z. (2021). Sense and sensibility: urban public signs during a pandemic, in Viral Discourse (Elements in Applied Linguistics), ed Jones R. H. (Cambridge: Cambridge University Press), 37–49.

[B15] Isosävi J. (in press). Finnish French public signs from commercial premises during the Covid-19 pandemic. Prag. Soc. Available online at: https://researchportal.helsinki.fi/en/publications/finnish-and-french-public-signs-from-commercial-premises-during-t.

[B16] Jakobson R. (1960). Linguistics and poetics, in Style in Language, ed Sebeok T. A. (Cambridge: MIT Press), 350–377.

[B17] Jing M. Wang M. X. (2021). Organizational communication and social mobilization - the spreading mode, discourse system and social governance function of public signs in fighting against the novel corona pandemic. J. Lover 6, 15–19.

[B18] Jørgensen A. Martinez J. A. (2010). Vocatives and phatic communion in Spanish teenage talk, in Love YA Hate YA: The Sociolinguistic Study of Youth Language and Youth Identities, ed Jørgensen J. N. (Cambridge: Cambridge Scholars Publishing), 193–209.

[B19] Kellaris J. J. Machleit K. Gaffney D. R. (2020). Sign evaluation and compliance under mortality salience: lessons from a pandemic. Interdiscipl. J. Sign. Wayfind. 4, 51–66. 10.15763/issn.2470-9670.2020.v4.i2.a65

[B20] Laver J. (1975). Communicative functions of phatic communion, in Organisation of Behaviour in Face-to-face Interaction, eds Kendon A. Harris R. M. Key M. R. (The Hague: Mouton), 215–238. 10.1515/9783110907643.215

[B21] Laver J. (1981). Linguistic routines and politeness in greeting and parting, in Conversational Routine: Explorations in Standardized Communication Situations and Prepatterned Speech, ed Coulmas F. (The Hague: Mouton Publishers), 289–304. 10.1515/9783110809145.289

[B22] Li L. (2020). Research of public slogans on epidemic prevention in the battle against COVID-19. J. Changzhi Univ. 37, 17–21.

[B23] Lyons J. (1968). Introduction to Theoretical Linguistics. London: Cambridge University Press. 10.1017/CBO9781139165570

[B24] Malinowski B. K. (1930) [1923]. The problem of meaning in primitive languages, in The Meaning of Meaning. A Study of the Influence of Language upon Thought and of the Science of Symbolism, eds Ogden C. K. Richards I. A. (New York, NY: Harcourt, Brace and Company, Inc.), 296–336.

[B25] Marshall S. (2021). Navigating COVID-19 linguistic landscapes in Vancouver's North Shore: official signs, grassroots literacy artefacts, monolingualism, and discursive convergence. Int. J. Multilingual. 10.1080/14790718.2020.1849225

[B26] Mautner G. (2012). Language, space and the law: a study of directive signs. Int. J. Speech Lang. Law 19, 189–217. 10.1558/ijsll.v19i2.189

[B27] McCarthy M. (2003). Talking back: 'small' interactional response tokens in everyday conversation. Res. Lang. Soc. Int. 36, 33–63. 10.1207/S15327973RLSI3601_3

[B28] Metslang H. (2004). Imperative and related matters in everyday Estonian. Linguist. Ural. 4, 243–256. 10.3176/lu.2004.4.02

[B29] Metslang H. Sepper M.-M. (2010). Mood in Estonian, in Mood in the Languages of Europe, eds Rothstein B. Thieroff R. (Amsterdam; Philadelphia, PA: John Benjamins Publishing Company), 528–550. 10.1075/slcs.120.29met

[B30] Ogiermann E. Bella S. (2021). On the dual role of expressive speech acts: relational work on signs announcing closures during the COVID-19 pandemic. J. Pragmat. 184, 1–17. 10.1016/j.pragma.2021.07.020

[B31] Orasmaa S. Petmanson T. Tkachenko A. Laur S. Kaalep H.-J. (2016). EstNLTK - NLP toolkit for Estonian, in Proceedings of the Tenth International Conference on Language Resources and Evaluation (LREC 2016). Available online at: https://estnltk.github.io/estnltk/1.4.1/

[B32] Pool R. (1999). About the use of different forms of the first and second person singular personal pronouns in Estonian cases, in Estonian: Typological Studies III (Publications of the Department of Estonian of the University of Tartu, 11), ed Erelt M. (Tartu: Tartu University Press), 158–184.

[B33] R Core Team (2021). R: A Language and Environment for Statistical Computing. Vienna: R Foundation for Statistical Computing. Available online at: https://www.R-project.org/

[B34] Rogers S. L. Howieson J. Neame C. (2018). I understand you feel that way, but I feel this way: the benefits of I-language and communicating perspective during conflict. PeerJ. 6, e4831. 10.7717/peerj.4831 29796350 PMC5961625

[B35] Sheikha F. A. Inkpen D. (2012). Learning to classify documents according to formal and informal style. Linguist. Issues Lang. Technol. 8, 1–29. 10.33011/lilt.v8i.1305

[B36] Shohamy E. (2006). Language Policy: Hidden Agendas and New Approaches. London; New York, NY: Routledge. 10.4324/9780203387962

[B37] Siewierska A. (2004). Person. Cambridge: Cambridge University Press. 10.1017/CBO9780511812729

[B38] Svennevig J. (2021). How to do things with signs. The formulation of directives on signs in public spaces. J. Pragmat. 175, 165–183. 10.1016/j.pragma.2021.01.016

[B39] Swales J. M. (1990). Genre Analysis. English in Academic and Research Settings. Cambridge: Cambridge University Press.

[B40] Tan M. S. Said S. B. (2015). Linguistic landscape and exclusion: an examination of language representation in disaster signage in Japan, in Conflict, Exclusion and Dissent in the Linguistic Landscape, eds Rubdy R. Said S. B. (London: Palgrave MacMillan), 145–169. 10.1057/9781137426284_7

[B41] Tragel I. Tomson K. (2022). Tanel ja Jüri kiusavad: piirangute põhjuste väljendusvahendid koroonapiirangute siltidel. / This is a whim of our government! Expressing the causes of the restrictions on COVID-19 signs. Õiguskeel 2, 1–10.

[B42] Wagner D. (2015). Öffentliche verbotsschilder in deutschland. Versuch einer typologie, in Kurze Texte und Intertextualität, eds Rink C. Skog-Södersved M. Reuter E. (Frankfurt am Main: Peter Lang), 123–138.

[B43] Wetzel P. J. (2010). Public signs as narrative in Japan. Jpn. Stud. 30, 325–342. 10.1080/10371397.2010.518601

[B44] Wierzbicka A. (1998). German 'cultural scripts': public signs as a key to social attitudes and cultural values. Discourse Soc. 9, 241–282. 10.1177/0957926598009002006

